# Supplementary material for: Improving follow-up survey completion rates through pilot interventions in the All of Us Research Program: Results from a non-randomized intervention study
Source: PLoS One. 2024 Oct 15;19(10):e0308995. doi: 10.1371/journal.pone.0308995 (PMC11478879; doi:10.1371/journal.pone.0308995)
Supplement: Attachment — Submitted filename: Reviewer Comments SCPI v2023-6-03.docx [file pone.0308995.s001.docx]

Reviewer Comments:

Reviewer #1: I appreciate the opportunity to review the manuscript titled “Improving Follow-Up Survey Completion Rates through Pilot Interventions in the All of Us Research Program" for PLOS ONE
The manuscript presents a reliable and comprehensive study to improve response rates for follow-up surveys. This study compared the response rates of three intervention group including telephone appointments, postal mail, and a combination of the two with control group that only received digital reminders. The study provides evidence that subpopulations have different response rates and reminders affect each subpopulation differently.
The manuscript is reliable both in terms of participant number and methodology, which makes it worth publishing.

**>>>>>>>>**

**Response: Thank you very much.**

**>>>>>>>>**

The only suggestion I have is to provide a little bit of information about the base-line and follow up questions and how much time does it take from participants.

**>>>>>>>>**

**Response: We added in the website where the questions are located to the Introduction:**

***The questions that participants answered are located here:*** [***https://www.researchallofus.org/data-tools/survey-explorer/***](https://www.researchallofus.org/data-tools/survey-explorer/)***.***

**We also added the following for mean (SD) completion times in the results**

***The baseline surveys and average completion times in minutes (mean, standard deviation) were: The Basics (6.69,3.48), Lifestyle (2.94, 1.65), and Overall Health (3.1, 1.32). The follow-up surveys and completion times were Health Care Access & Utilization (6.41, 2.72), Personal Health History (7.15, 3.88 ), and Family Health History (6.61, 3.90).***

**>>>>>>>>**

Reviewer #2: The study describes a non-randomised study of interventions that compared three inventions aimed at increasing response rates for follow-up surveys in the all of us research program. The interventions consisted of postal or phone reminders. The main problem of the study is that individuals were not randomly allocated to the conditions limiting so the interpretation of the results due to the potential biases. Additionally, the introduction, methods and discussion sections miss relevant literature. There are no previous studies mentioned that tested reminders or the effect of offering an alternative participation modality. The results of the study are also not discussed in line with previous studies.

Title:
• The title should state that is not a randomised study. Something along the lines of: “Improving Follow-Up Survey Completion Rates through Pilot Interventions in the All of Us Research Program: Results from a non-randomised intervention study”

**>>>>>>>>**

**Response: We changed the title to the following:**

***Improving Follow-Up Survey Completion Rates through Pilot Interventions in the All of Us Research Program: Results from a non-randomized intervention study***

**>>>>>>>>**

Abstract:
• In general, the abstract is very short and missing some important information. The limit for the word count should be 300 words, but the authors only used 188. The missing information were:
o Telephone appointment intervention could be briefly explained in brackets. You should mention that it consists of offering an alternative participation modality.

o The digital online reminder could also be explained. It sounds like an email reminder.

**>>>>>>>>**

**Response: We added a brief description of the telephone intervention, postal, and digital only reminder in brackets:**

***The three arms were: (1) telephone appointment [staff members calling participants offering appointments to complete surveys over phone] (2) postal [mail reminder to complete surveys through U.S. Postal Service], and (3) combination of telephone appointment and postal. Controls received digital-only reminders [program-level digital recontact via email or through the participant portal].***

**>>>>>>>>**

o If the word count allows it, it would be great to get some information about the allocation to the conditions (stating that it was not random).

**>>>>>>>>**

**Response: We added the following about allocation to study arms:**

***Study sites chose their study arm and participants were not randomized***

**>>>>>>>>**

o Absolute completion rates should be communicated. What was the baseline?

**>>>>>>>>**

**Response: We added the absolute completion rates of follow-up surveys. All of these participants completed baseline surveys.**

***Of all participants, 6,828 (10.4%) completed any follow-up surveys (1448 : telephone; 522: postal; 486: combination; 4372: controls). Follow-up survey completions were 24% higher in the telephone appointment arm than in controls in bivariate analyses.***

**>>>>>>>>**

o The conclusion sounds very general and only highlights that the study improved understanding of how to improve retention rates. Nothing was said about the implications that telephone appointments were the only successful intervention (alone or in combination with the postal reminder

**>>>>>>>>**

**Response: We added that telephone appointments were the most successful intervention to the conclusions.**

***Telephone appointments appeared to be the most successful intervention in our study.***

**>>>>>>>>**

Introduction:
• The introduction section is very short and missed some information from previous literature on why individuals don’t fill out follow-up surveys.

**>>>>>>>>**

**Response: We added some information from previous literature and why individuals don’t fill out follow-up surveys**

***There are multiple reasons why individuals don’t fill out follow up surveys including issues with accessing or submitting the survey, technical issues, not receiving messages, lack of interest, survey being boring or too long, or no time or bad timing[1].***

**>>>>>>>>**

• There is no rationale for the tested interventions. There surely must been studies that have tested these interventions beforehand.

**>>>>>>>>**

**Response: We clarified this in our introduction as these interventions have been effective in prior studies.**

***Prior studies have shown that interventions, such as phone calls, postal mailings, or both have improved follow-up survey completion rates[2-7]. This extensive literature on survey methods shows that multimodal survey methods are more effective than single-mode methods.***

**>>>>>>>>**

• How do you define the underrepresented population? What are the characteristics?

**>>>>>>>>**

**Response: the definition is described in our prior work[8]:**

**However we added a sentence that further describes the definition and added the reference:**

***For underrepresented populations, All of Us relied on designated definitions of diversity guided by the leading authorities on health disparities and through consultation with a variety of resources and stakeholders as described in our prior work[8]***

**>>>>>>>>**

• It would also have been helpful to get some information/theory/model about what the interventions target specifically (e.g. what is the rational that telephone appointments should be more effective that postal reminder)? They both seem to address the problem of procrastination, but the phone is more difficult to avoid.

**>>>>>>>>**

**Response: We added the following to the manuscript**

***There is a scarcity of both theoretical and practical guidance on crafting optimal surveys for mixed-mode data gathering, such as utilizing both telephone and postal methods[9]. Nevertheless, survey creators often opt for a mixed-mode strategy because it allows for mitigating the drawbacks of each mode while maintaining affordability. By blending a primary method with a secondary, pricier one, researchers can benefit from reduced costs and errors compared to a single-mode approach. Mixed-mode designs entail a deliberate balance between expenses and inaccuracies, particularly addressing non-sampling errors like frame or coverage error, nonresponse error, and measurement error[9].***

**>>>>>>>>**

Method
• The methods section is also very short and should feature more information about the study design.
o The study nature (non-randomised intervention study) should be mentioned in the design section.

**>>>>>>>>**

**Response: We added the following to the design section:**

***Study sites chose their study arm and participants were not randomized.***

**>>>>>>>>**

o I think that the telephone appointment intervention is kind of complex and requires some more details as it does not only remind individuals about the survey but also offers them to fill it out over the phone (CATI), which may be more convenient for them. Some literature on the perception of CATI would help the reader to better understand that it was not just a reminder, but the offer of a different modality.

**>>>>>>>>**

**Response: We clarified the telephone appointment intervention with the following:**

***The telephone appointment not only reminded individuals about the survey but also offered them the opportunity to fill the surveys out over the phone, like computer assisted telephonic interviews (CATI), which may be more convenient for them. CATI refers to a method of conducting surveys or interviews over the telephone using a computer program to assist interviewers in the process. The software typically helps with tasks such as questionnaire administration, data entry, and management, increasing the efficiency and accuracy of the interview process*** ***[10].***

**>>>>>>>>**

o The combination arm should also be explained in more detail. What were the timepoints of the two interventions? Were individual first sent the letter and then called? If yes, then the letter serves a primer. This should be acknowledged.

**>>>>>>>>**

**Response: We clarified the combination arm as follows:**

***The combination arm consisted of an initial contact via a phone call followed by a postal mailing if the participant had not completed a survey within thirty days of the initial call. The initial calls were made at times when staff were able, and not at a specific time period during the pilot implementation. The postal mailing included the Introductory letter and the Survey Instruction Brochure to facilitate portal access for survey completion.***

**>>>>>>>>**

o Some information about the study population would be welcome. Apart from the sentence in the introduction about the All of Us study, nothing is said about the characteristics of the study population, such as age range, …

**>>>>>>>>**

**Response: We added the following verbiage and reference for the all of us study population to the “Identification of participants in the Intervention and Control Groups” section.**

***The All of Us Research Program aims to enroll a diverse group as described in the introduction of at least one million people living in the United States, regardless of health status, age, race, ethnicity, sexual orientation, gender identity, or socioeconomic status. The goal is to gather health data from a wide range of individuals to better understand how genetics, lifestyle, environment, and other factors contribute to disease and overall health[11].***

**>>>>>>>>**

o What happened to study participants, who missed more than one follow-up survey. Were they asked to fill out just one or all of them?

**>>>>>>>>**

**Response: Participants were asked to fill out all surveys. We added this to the “Controls and Interventions” section:**

***Participants in all arms and controls were asked to fill out all surveys.***

**>>>>>>>>**

Analysis section
o You mentioned that study participants needed to have missed at least one follow-up survey to qualify for the intervention, thus the outcome measure is completing any missing follow-up survey. The completion rate is therefore just reefing to completing at least one follow-up. This needs to be described clearly.

**>>>>>>>>**

**Response: For clarity we clarified and moved the following to the beginning of the paragraph of the analysis section:**

***The study analysis aimed to determine the following: (1) whether completion rates of at least one follow-up survey increased in one or more study arms; (2) whether at least one follow-up survey completion rates increased more in the telephone appointment arm than the postal arm; and (3) whether at least one follow-up survey completion rates increased more in the combination arm than the other two arms.***

**>>>>>>>>**

o Could you also include the number of missing follow-up surveys as a covariate in the regression model? It would be nice to see if the interventions’ impact is associated with that.

**>>>>>>>>**

**Response: Yes, we made this modification and added this covariate to the models. The methods were updated as below as were results and figures to include these covariates:**

***These covariates included the demographic variables listed above, intervention study arm, Social Security number question missingness and not providing an email address (as proxies for participant engagement), health literacy score from the Brief Health Literacy Scale (which is part of the All of Us Overall Health Survey), time of enrollment since All of Us initiation (in weeks), number of missing follow-up surveys, and enrollment site size (total number of participants at the site).***

**>>>>>>>>**

o Did the regression control for that clustering of the intervention? In the method section it is written, that individuals were allocated to arms according to the preferences of the sites. You should control for this.

**>>>>>>>>**

**Response: In this revision, the site was incorporated into the model as a random effect and results were updated to reflect the change.**

**>>>>>>>>**

Results:
• Please include a table of the characteristics of the study sample according to the conditions and also conduct some univariate tests to see if there are differences in the allocation. This is to check for selection biases. I believe that you put it in the appendix (table 1). If this is the case, then mention the results of the tests, it does not seems as if the conditions were balanced. Please also refer to that table in the results section and not just the discussion.

**>>>>>>>>**

**Response: We agree. The conditions were not balanced in the univariate analysis, and we have incorporated this observation into the Results section. As per your suggestion in the subsequent comment, we have included detailed information regarding the propensity score model in the Method section, along with a figure comparing the propensity scores of the intervention and control arms, now situated in the Results section. Our findings indicate that although there are discrepancies in the balance of individual conditions between the intervention and control arms, their propensity score distributions exhibit significant overlap. Consequently, we assert that a multivariable regression model, adjusting for these conditions, would be appropriate for comparing the intervention arms to the controls.**

**This is the propensity score figure.**

**>>>>>>>>**

• Provide also some descriptive statistics about the characteristics of the overall study participants in text form

**>>>>>>>>**

**Response: We added some descriptive statistics about the characteristics of the overall study participants in text form:**

***Of the total number of participants (65,425) in the intervention and control groups, 20,427 (30.9%) were White, 1,628 (2.4%) Asian, 25,615 (39.2%) Black or African American, 11,114 (17%) Hispanic, Latino or Spanish, and 6,641 (10.1%) did not provide racial and ethnic information. There were 10,056 (15.4%) participants had less than high school education, 16,947 (25.9%) with high school education, 16,307 (24.9%) had some college education, 19,384 (29.6%) with a college degree, 2,731 (4.1%) did not provide education information. 23,139 (35.4%) participants were between age 18-45, 29,466 (45%) between age 45-65, 9272 (14.2%) between age 65-75 and 3,548 (5.4%) participants older than 75. 6,828 (10.4%) completed any of the follow-up surveys, and of these, 6,725 (98.5%) completed all three follow-up surveys.***

**>>>>>>>>**

• Mention the completion rates in absolute terms in the results for all conditions. The figures are of low resolution and difficult to read. You could easily translate them into tables to make it easier to understand.

**>>>>>>>>**

**Response: We added the completion rates to a part of the figures to improve the clarity as mentioned above.**

**>>>>>>>>**

• What was the rational for the interaction analysis? If this is based on findings from previous literature, then you should add a reference in the introduction section and state that you want to look at it too.

**>>>>>>>>**

**Response: The interaction analysis stemmed from a stratified comparison of completion rates between the intervention and control arms (Fig 3). We observed significant disparities between underrepresented and represented groups in biomedical research. The two groups were defined by race/ethnicity, sex at birth, gender, age, education, household income, and geographical residence. Consequently, we investigated the potential interaction effects of these individual factors.**

**In this revision, we added the following under Descriptive and Multivariable Analyses (renamed to Statistical Analysis)**

***Given the All of Us program's emphasis on enrolling underrepresented populations, we also stratified the incremental completion rates by underrepresented (UBR) and represented (RBR) groups[8].***

***Descriptive statistics showed different intervention effects between UBR and RBR groups, highlighting the need to study interactions between intervention arms and characteristics included in the UBR definition variables.***

**>>>>>>>>**

Discussion
• Overall the discussion is missing relevant literature and the implications of the imbalances in the conditions should be discussed in more detail. For me the simple regression does not make much sense in this context and could be removed completely.

**>>>>>>>>**

**Response: We have addressed the reviewer's comments by providing additional details, following your recommendation to include comprehensive information about the propensity score model in the Method section and incorporating a figure comparing the propensity scores of the intervention and control arms into the Results section. Our analysis revealed that although there were discrepancies in the distribution of individual conditions between the intervention and control arms, there was considerable overlap in their propensity score distributions. Thus, we advocate for the utilization of a multivariable regression model to compare the intervention arms to the controls, as it effectively controls for these conditions.**

**The regression analysis underscores the effectiveness of the intervention arms while controlling for covariates, which is crucial given the potential biases inherent in the study if covariates are not adequately controlled for. Moreover, we acknowledge that while other propensity score-based methods such as propensity score matching or weighting are suitable for addressing condition imbalances in observational studies like ours, they are not conducive to the interaction analysis, which is a significant aspect of our study. Therefore, we opted to assess the overlap in propensity score distributions to identify potential confounders and employed a multivariate regression model to adjust for these confounders, as well as to explore potential moderator effects.**

**>>>>>>>>**

o You could add some links to findings from previous studies that tested similar interventions.

o How do your results align with them and how does your study differ in terms of interventions, study population and context.

**>>>>>>>>**

**Response: We added the following to the discussion to describe findings from previous studies that tested similar interventions and discussed how our results align with them and how does your study differ in terms of interventions, study population and context**

***A systematic review by Booker et al.[12] of retention methods in population-based cohort studies described strategies, such as reminders, repeat contacts (phone or mail), and repeated mailings of questionnaires were reviewed for 17 studies showing increases in survey response rates of 2%–37%. Higher numbers of reminder letters or postcards led to higher response rates; one study showed increased response rates for mailing a second questionnaire compared with a postcard. Using multiple retention methods including phone calls increased survey response rates by more than 70%. Our results showed increases of up to 24% higher with phone calls. Our lower rates of improvement could be because of the scale of the program or the population that may be more challenging to retain.***

**>>>>>>>>**

• Can elaborate on this statement: “If sites focused on fewer participants who would be less likely to complete additional modules because they were less digitally literate, the digitally literate participants might have completed follow-up surveys in the control arm.”

**>>>>>>>>**

**Response: We modified the sentence to say the following:**

***If sites focused on fewer participants who would be less likely to complete additional survey modules because they were less digitally literate in any one of the intervention arms, it is possible that more of the digitally literate participants might have completed follow-up surveys in the control arm.***

**>>>>>>>>**

• Can you cite studies that tested postal reminders for online survey and what they found?

**>>>>>>>>**

**Response: We added this as response to the above**

**>>>>>>>>**

• Can you say something more about including incentives? Are they monetary, conditional, lottery-based,…

**>>>>>>>>**

**Response: We added some details about incentives**

***All of Us partners have begun to pilot incentives to engage participants in follow-up surveys. These incentives varied and included five dollars per follow-up survey completed, thirty dollars for completion of all three follow-up surveys within a four-week period, and different amounts given to complete them within certain timeframes.***

**>>>>>>>>**

• You should mention propensity score in the statistical analysis section already.

**>>>>>>>>**

**Response: We agree. Following your suggestion, we have included detailed information regarding the propensity score model in the Method section, along with a figure comparing the propensity scores of the intervention and control arms, now situated in the Results section. Our findings indicate that although there are discrepancies in the balance of individual conditions between the intervention and control arms, their propensity score distributions exhibit significant overlap. Consequently, we assert that a multivariable regression model, adjusting for these conditions, would be appropriate for comparing the intervention arms to the controls.**

**>>>>>>>>**

**REFERENCES**

1.	Couper MP, Peytchev A, Strecher VJ, Rothert K, Anderson J. Following up nonrespondents to an online weight management intervention: randomized trial comparing mail versus telephone. J Med Internet Res. 2007;9(2):e16. Epub 2007/06/15. doi: 10.2196/jmir.9.2.e16. PubMed PMID: 17567564; PubMed Central PMCID: PMCPMC1913938 which developed and has proprietary interest in the tailored weight management program described herein.

2.	Dillman DA, Smyth JD, Christian LM. Internet, phone, mail, and mixed-mode surveys: The tailored design method: John Wiley & Sons; 2014.

3.	Couper MP. New developments in survey data collection. Annual Review of Sociology. 2017;43:121-45.

4.	Dillman DA. The promise and challenge of pushing respondents to the web in mixed-mode surveys: Statistics Canada; 2017.

5.	DeLeeuw ED, editor Mixed-mode: Past, present, and future. Survey Research Methods; 2018.

6.	Freedman VA, McGonagle KA, Couper MP. Use of a targeted sequential mixed mode protocol in a nationally representative panel study. Journal of Survey Statistics and Methodology. 2018;6(1):98-121.

7.	Patrick ME, Couper MP, Laetz VB, Schulenberg JE, O’Malley PM, Johnston LD, Miech RA. A sequential mixed-mode experiment in the US National Monitoring the Future Study. Journal of survey statistics and methodology. 2018;6(1):72-97.

8.	Mapes BM, Foster C.S., Kusnoor, S.V., Epelbaum, M.I., AuYoung M., Jenkins G., Lopez-Class, M., Richardson-Heron D., Elmi, A., Surkan, K., Cronin, R.M., Wilkins, C.H., Perez-Stable, E.J., Dishman, E., Denny, J.C., Rutter, J.L. Diversity and Inclusion for the All of Us Research Program: A Scoping Review. In: RM C, editor. 2020.

9.	De Leeuw D. To mix or not to mix data collection modes in surveys. Journal of official statistics. 2005;21(2):233.

10.	Peterson R, Hedden SL, Seo I, Palacios VY, Clark EC, Begale M, et al. Rethinking Data Collection Methods During the Pandemic: Development and Implementation of CATI for the All of Us Research Program. J Public Health Manag Pract. 2024;30(2):195-9. doi: 10.1097/phh.0000000000001846. PubMed PMID: 38271102; PubMed Central PMCID: PMCPMC10827348.

11.	Denny JC, Rutter JL, Goldstein DB, Philippakis A, Smoller JW, Jenkins G, Dishman E. The "All of Us" Research Program. N Engl J Med. 2019;381(7):668-76. Epub 2019/08/15. doi: 10.1056/NEJMsr1809937. PubMed PMID: 31412182.

12.	Booker CL, Harding S, Benzeval M. A systematic review of the effect of retention methods in population-based cohort studies. BMC Public Health. 2011;11:249. Epub 2011/04/21. doi: 10.1186/1471-2458-11-249. PubMed PMID: 21504610; PubMed Central PMCID: PMCPMC3103452.
